# Prevalence of Overweight and Obesity among Primary School Children Aged 8–13 Years in Dar es Salaam City, Tanzania

**DOI:** 10.1155/2016/1345017

**Published:** 2016-06-14

**Authors:** Ismail N. Pangani, Festus K. Kiplamai, Jane W. Kamau, Vincent O. Onywera

**Affiliations:** ^1^Department of Physical Education and Sport Sciences, University of Dar es Salaam, P.O. Box 35048, Dar es Salaam, Tanzania; ^2^Department of Recreation Management and Exercise Science, Kenyatta University, P.O. Box 43844, Nairobi 00100, Kenya

## Abstract

*Background*. The understanding of obesity as a growing health problem in Africa and Tanzania in particular is hampered by lack of data as well as sociocultural beliefs in which overweight and obesity are revered. This study sought to determine the prevalence of overweight and obesity among primary school children aged 8–13 years in Dar es Salaam, Tanzania.* Method*. A cross-sectional analytical research design was used to study overweight and obesity in primary schools in Dar es Salaam, Tanzania. The target population was 150,000 children aged 8–13 years. Stratified random sampling was used to select 1781 children. Weight and height were taken and WHO standards for children were used to determine weight status.* Results*. Findings showed that the prevalence of overweight and obesity was 15.9% and 6.7%, respectively (*N* = 1781). However, 6.2% of the children were underweight. There were significant differences in mean BMI between children in private and public schools (*p* = 0.021), between male and female (*p* < 0.001), and across age groups of 8–10 and 11–13 years (*p* < 0.001).* Conclusion*. The prevalence of overweight and obesity among primary school children is significant and requires management and prevention strategies.

## 1. Introduction

Childhood obesity has more than tripled worldwide in the last 30 years [1]. The prevalence of obesity among children aged 6 to 11 years increased from 6.5% in 1980 to 19.6%, while, in the same years, that of children in the age group of 12 to 19 increased from 5.0% to 18.1% in 2008 [2]. Once considered a problem only in high-income countries, childhood overweight and obesity are on the rise in low and middle income countries, particularly in urban settings that are currently faced with physical activity and nutrition transition. This has led to overconsumption of energy dense foods with reduced participation in physical activity [3–5]. In developing countries such as India, especially in urban populations, childhood obesity is emerging as a major health problem [6]. Therefore, the obesity epidemic is not restricted to industrialized societies because studies show that the increase in obesity cases is even faster in developing countries than in the developed world [7]. Often coexisting with malnutrition in developing countries, overweight and obesity are complex conditions, with serious social, physiological, and psychological dimensions, affecting all age and social economic groups [8]. It is evident that the rapid urbanization, technology, and the dramatic lifestyle changes experienced by people in African cities are among risk factors of the overweight and obesity epidemic [9]. It is indicated that the impact of urbanization on lifestyle is likely to be reflected in urban adolescents' lower level of physical activity and higher indices of adiposity than of the rural counterparts [10]. Studies demonstrate that overweight children tend to become overweight adults [11]. This suggests that effective prevention and management of overweight and obesity in the society are best encountered by preventing and managing the epidemic in childhood [12]. A study conducted by Mosha and Fungo [13] found that there was limited knowledge on obesity and its health consequences among primary school children in Dar es Salaam, Tanzania. Another study [14] conducted in Kinondoni, Dodoma, found that 5.6% and 6.3% of children aged 6–9 were overweight and obese, respectively. However, the effect of urbanization and physical activity transition in Tanzania's largest city Dar es Salaam on the prevalence of overweight and obesity has not been studied, hence this study. Therefore, the study sought to assess the prevalence of overweight and obesity among primary school children aged 8–13 years in Dar es Salaam City in Tanzania.

## 2. Methodology

This study used a cross-sectional analytical research design. Research Permits from the Ministry of Education and Vocational Training (MoEVT), from the Regional Administrative Secretary and Districts' Administrative Secretaries, were obtained. School heads were contacted in person for permission and convenience of the study process. Parents were given informed consent one week before data collection from the school where their children were enrolled. Assent letters were given to the children and asked to freely declare participating or not participating in the study. The study targeted 150,000 children aged 8–13 years attending grades 4 to 6 in both government and private primary schools in Dar es Salaam City, Tanzania.

### 2.1. Exclusion Criteria

The following were excluded from the study:

- Children who were within the age group of the study population but were not in classes/grades 4 to 6.
- Children older than 13 years.

The sample comprised 1,781 school children that were obtained by stratified, random, and quota sampling methods. Stratification was conducted based on location, type of school, and gender where each of the three districts, Kinondoni, Ilala, and Temeke, formed subpopulations. In each district, private schools and public schools and inside schools, male and female pupils formed strata from which study participants were randomly selected. The study used BMI on percentile ranks as a criterion to classify children into the predetermined body-weight categories, namely, underweight, normal/healthy weight, overweight, and obese. Children with BMI within the 5th percentile or below were classified underweight; those with BMI above the 5th but below the 85th percentiles were classified as normal/healthy, while those between the 85th and 95th percentiles were classified as overweight and the ones above the 95th percentile were classified as obese. Therefore, the height and weight measurements were taken and used to compute for the children's BMI which were then translated into weight status using the World Health Organization age and sex specific percentile ranks [4]. The study involved 32 schools, of which 12 were private while 20 were government owned. There were 1028 (58%) females and 753 (42%) males. The average age of the participants was 10.5 ± 2.5 years.

### 2.2. Data Collection

Data were collected from July to October 2012. The researcher and three research assistants visited all the sampled schools from Monday to Friday in a week during school hours and each school was visited for not more than three times. In the first visit to each school, children were sampled and given consent letter for their parents or guardian; this visit took place one week before the second visit in which data were collected. On arrival in the second visit, consent letters from the parents/guardian were collected from the children and those who were allowed to participate in the research were gathered in class and given assent forms to read and sign upon their acceptance before the measurement of anthropometric and the filling of questionnaires started.

### 2.3. Stature

Height was measured using stadiometer (Leicester-21400, CECA, UK). Participants were asked to remove shoes and headscarf and undo their hairstyle and hairstyle accessories (where applicable) before stepping on stadiometer placed on a flat floor along the wall. The pupils were advised to keep their heels together, feet flat on the stepping board of the stadiometer, and to inhale deeply, hold the breath, and maintain an erect anatomical posture [14]. The head was positioned in such a way that the angle of the eye and the opening of the external auditory meatus were in a horizontal line [14]. Height measurement was then carefully read to the nearest 0.1 cm.

### 2.4. Weight

Weight was determined using a digital weighing scale (7841-Medscale Bluetooth, SOEHNLE). Measurements were taken with each pupil in light clothing and without shoes and socks [14]. Weight was carefully read when the point readings stabilized and was recorded to the nearest 0.1 kg.

### 2.5. Data Analysis

Data obtained were analyzed, coded, and entered into Statistical Package for Social Sciences (SPSS) program version 19.0. Frequencies, means, percentages, and standard deviations were calculated and presented in tables, while inferential statistics were tested by an independent *t*-test and a *p* value < 0.05 was considered significant in the testing of hypotheses.

### 2.6. Findings

The study found that most (71.3%) of the participants were in the normal weight category while 15.9% were overweight and 6.7% were obese while 6.2% were underweight. The results also showed that overweight and obesity were more prevalent among children in the lower age group of 8–10 years than in age group of 11–13 years.

Table 1 shows that 5.5%, 18.7%, and 8.0% of females and 7%, 12.1%, and 4.9% of males were underweight, overweight, and obese, respectively. This showed that more males were found to be underweight (7%) than females (5.5%), while more females were found to be overweight or obese (26.7%) than males (17%).

To find out if the difference in prevalence of overweight and obesity was significant among age groups, type of school, and sex, independent *t*-tests were conducted and results are presented in Table 2.

The results in Table 2 indicate that children aged between 8 and 10 years were more overweight and obese (M = 18.02, SD = 3.11) than those aged 11–13 years (M = 18.4, SD = 4.35) (*p* < 0.001). Children from private schools were more overweight and obese (M = 18.41, SD = 4.57) than their counterparts in public schools (M = 17.94, SD = 3.4) at *p* = 0.021. Similarly, females were more overweight or obese (M = 18.50, SD = 4.89) than males (M = 17.61, SD = 2.84) at *p* < 0.001. Therefore, children in private schools, females, and younger age groups of 8–10 years showed a higher prevalence to overweight and obesity than their counterparts in public schools, males, and age groups of 11–13 years.

## 3. Discussions

Obesity is no longer restricted to developed countries [15]. The trends of overweight incidences in school children tend to increase over time [16]. Previous studies in Tanzania showed that the prevalence of overweight and obesity combined among children aged 6–9 years in Dodoma and Kinondoni was 9.8% and 14.9%, respectively [13]. For children aged 10–12 years, the prevalence of overweight and obesity was 8.8% in Dodoma and 11.6% in Kinondoni municipalities [13]. The present study found that the prevalence of overweight and obesity combined in Dar es Salaam was 22.6%. Additionally, 6.2% of the respondents were found to be underweight. In a similar regional study which used objective measurements of adiposity in Nairobi County in Kenya, it was reported that, among the primary school children in the province, 6.7% and 20.2% were underweight and overweight or obese, respectively [17].

Another study conducted in Nigeria identified the prevalence of overweight, obesity, and thinness among children aged 5 to 18 years to have been 11.4%, 2.8%, and 13.0%, respectively [18]. Developing countries therefore face the double burden of obesity and undernutrition [19]. It is perceived in African settings that fatness with rounded body shapes among females is an attribute of beauty [20]. It follows that female children in such setting do not feel disadvantaged when they become overweight. The results from this study found out that the mean BMI of the male children was lower (M = 17.61, SD = 2.84) than that of females (M = 18.50, SD = 4.89) and the difference was statistically different at *p* < 0.001.

This implied that females were more likely to be overweight or obese than male counterparts. The findings of this study were similar to that obtained from other regional studies that females were more overweight or obese than males. A study on primary school children in Nairobi, Kenya, reported higher prevalence of overweight and obesity among females (10.9% and 3.6%) than males (6.5% and 2.6%), respectively [21]. In another study, though males and females did not differ in terms of adiposity, females were more overweight than males [17]. In another study on children aged 9 to 13 years in western Kenya [22], it was found that 6.8% of the boys and 16.7% of the girls in the urban schools were overweight and/or obese. The findings also are in line with that found in South African children where there was higher prevalence of overweight and obesity among female pupils 17.9% and 4.9% than among boys 14.0% and 3.2%, respectively [23]. In another study among rural South African children, it was reported that the prevalence of underweight was significantly higher in boys than in girls [24]. Similarly, some distant studies report a higher prevalence of overweight and obesity among females than in males. For example, one study reported higher prevalence of overweight and obesity among females (28%) than males (23%) in Ireland health survey [25]. In concurrence with the present findings, a study among 5–14-year-old children in India found that male children were thinner than female children [26].

Although the findings of this study gave a regional consistence of the prevalence of overweight and obesity being higher in females than males, there are some studies in contrast. The findings of this study differed with those of Australian O'Dea in 2008 and that of Iranian Hajian-Tilaki in 2011 who found that male children showed more prevalence in obesity than the females. A study by O'Dea found that the prevalence of obesity among Australian primary school boys was higher (6.4%) than girls (5.6%) [27]. In line with O'Dea, Hajian-Tilaki found that Iranian males had higher overweight and obesity prevalence (10.9% and 3.6%) than females (6.5% and 2.6%), respectively [28]. Studies in Denmark and in Tonga reported higher prevalence of overweight and obesity among males than females in primary schools [29, 30]. While in Denmark the prevalence of 29.3% in males and 21.1% among females was reported [29], in Tonga the prevalence of the same was 25% in males and 18% among females [30]. The inconsistence in the findings among studies may be explained by differences in the study populations. It is stated that the demography of obesity prevalence differs with culture, structure, and ecology of the environment of the subjects [31]. Therefore, the incidences of obesity among males and females differ greatly with differences attributed to cultural factors [32]. This may be the reason why regional studies produced similar results but differed with distant studies.

The present study showed that lower age groups (age 8–10 years) were more likely to be overweight or obese than age group of 11-12 years. Concurrently, worldwide studies showed that the prevalence of overweight and obesity combined among children in age of 6 to 11 years increased from 6.5% in 1980 to 19.6% in 2008, while in children in age group of 12 to 19 the prevalence increased from 5.0% to 18.1% in 2008 [2]. These findings suggest that the prevalence of overweight and obesity is on the rise. The possible explanations can be attributed to the increased sophistication of life style to which new generations are exposed.

Incidences of overweight and obesity are more common in the middle and high cost private schools than in public schools [17]. The findings of this study revealed that the prevalence of overweight and obesity was higher among private school children 22.1% and 11.4% than their counterparts in public primary schools 12.1% and 3.8%, respectively. In agreement with these findings, a study in Kenya reported that private school children in Nairobi province were found to be 5.1 times more likely to be overweight/obese than their counterparts in public school [17]. The study further revealed that public school children were more likely to be underweight than those in private schools. This finding was in line with those of a study in western Kenya which reported that there was higher prevalence of overweight/obesity among the participants from private schools compared to their counterparts from the public schools [21, 33, 34]. Most of the underweight children in Dar es Salaam reported during interview to have missed some meals suggesting that the lower weight was a result of undernutrition. Similarly, it was reported by another study that there was an increase of obesogenic environment to children who were restricted in movement by their parents or guardians; this is even more evident in children who have less and less opportunities to involve in spontaneous physical activities [31].

Although the prevalence of overweight and obesity was not high by the time this study was conducted as majority (71.3%) were of normal weight, the trends showed an increase with time that needs attention. The prevalence of overweight and obesity combined among children aged 6–9 in Kinondoni, Dar es Salaam (*N* = 71), was 14.9% in 2009 [13], which had increased as for this study to 22.6%. In line with the findings from this study, a quantitative synthesis revealed a trend towards increasing proportions of overweight/obesity over time in school-aged children in Sub-Saharan Africa, as well as a persistent problem of underweight [16]. It is therefore imperative for the public to consider the trends as an indicator of a growing double edged burden where children who have enough food are prone to overweight and obesity while those who do not have enough food are prone to underweight.

## 4. Conclusion

The prevalence of overweight (15.9%) and obesity (6.7%) among primary school children in Dar es Salaam is high which needs deliberate intervention strategies. Paralleled with obesity is the underweight: 6.2% of the study children were found to be underweight. It is therefore important to consider that while addressing strategies on how some children can lose weight some need to put on more weight.

## Figures and Tables

**Table 1:** Prevalence of underweight, overweight, and obesity among primary school children aged 8–13 years in Dar es Salaam (*N* = 1781).

Prevalence	*N*	Underweight	Normal weight	Overweight	Obese
By age group					
Age of 8–10 years	939	2.5%	64%	24%	9.5%
Age of 11–13 years	842	9.9%	78.6%	7.8%	3.9%

Prevalence by sex					
Male	753	7.0%	76.0%	12.1%	4.9%
Female	1028	5.5%	67.8%	18.7%	8.0%

By type of school					
Private	678	3.2%	63.3%	22.1%	11.4%
Public	1103	8.0%	76.2%	12.1%	3.8%

Total	1781	6.2%	71.3%	15.9%	6.7%

**Table 2:** Descriptive data and *t*-tests of the mean BMI by age, type of school, and sex among primary school children aged in Dar es Salaam (*N* = 1781).

	*n*	Mean BMI	SD	*p* value
By age group				
8–10	939	18.02	3.11	0.001
11–13	842	18.41	4.35	

By type of school				
Public	1103	17.94	4.57	0.021
Private	678	18.00	3.41	

By sex				
Female	1028	18.50	4.89	0.001
Male	753	17.61	2.84	
